# Molecular mechanisms of chemo‐ and radiotherapy resistance and the potential implications for cancer treatment

**DOI:** 10.1002/mco2.55

**Published:** 2021-06-10

**Authors:** Ya‐Ping Liu, Can‐Can Zheng, Yun‐Na Huang, Ming‐Liang He, Wen Wen Xu, Bin Li

**Affiliations:** ^1^ MOE Key Laboratory of Tumor Molecular Biology and Key Laboratory of Functional Protein Research of Guangdong Higher Education Institutes Institute of Life and Health Engineering Jinan University Guangzhou P. R. China; ^2^ MOE Key Laboratory of Tumor Molecular Biology and Guangdong Provincial Key Laboratory of Bioengineering Medicine National Engineering Research Center of Genetic Medicine Institute of Biomedicine College of Life Science and Technology Jinan University Guangzhou P. R. China; ^3^ Department of Biomedical Sciences City University of Hong Kong Hong Kong China

**Keywords:** cancer, chemoresistance, molecular mechanisms, radioresistance, targeted therapy

## Abstract

Cancer is a leading cause of death worldwide. Surgery is the primary treatment approach for cancer, but the survival rate is very low due to the rapid progression of the disease and presence of local and distant metastasis at diagnosis. Adjuvant chemotherapy and radiotherapy are important components of the multidisciplinary approaches for cancer treatment. However, resistance to radiotherapy and chemotherapy may result in treatment failure or even cancer recurrence. Radioresistance in cancer is often caused by the repair response to radiation‐induced DNA damage, cell cycle dysregulation, cancer stem cells (CSCs) resilience, and epithelial‐mesenchymal transition (EMT). Understanding the molecular alterations that lead to radioresistance may provide new diagnostic markers and therapeutic targets to improve radiotherapy efficacy. Patients who develop resistance to chemotherapy drugs cannot benefit from the cytotoxicity induced by the prescribed drug and will likely have a poor outcome with these treatments. Chemotherapy often shows a low response rate due to various drug resistance mechanisms. This review focuses on the molecular mechanisms of radioresistance and chemoresistance in cancer and discusses recent developments in therapeutic strategies targeting chemoradiotherapy resistance to improve treatment outcomes.

## Graphical abstract

This review discussed the molecular mechanisms of cancer resistance to radiotherapy and chemotherapy including enhanced DNA damage repair, reduced intracellular accumulation of drugs, drug inactivation, changes in drug targets, apoptosis‐growth balance disruption. We also focused on the roles of oncogenes and tumor suppressor genes in therapeutic resistance. Some anticancer drugs targeting oncogenes associated with therapeutic resistance were summarized.

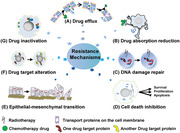

## 1 INTRODUCTION

Cancer, a global challenge that threatens human health, sees approximately 18.1 million new cases annually, with 9.6 million cancer‐related deaths reported in 2018.^1^ For both sexes, lung cancer is the most commonly diagnosed cancer (11.6% of the total cases) and the leading cause of cancer‐related death (18.4% of the total cancer deaths), closely followed by breast cancer in women (11.6%), colorectal cancer (CRC) (10.2%), and prostate cancer (7.1%) for incidence and CRC (9.2%), stomach cancer (8.2%), and liver cancer (8.2%) for mortality.^1^ Cancer is mainly treated with a combination of surgery, chemotherapy, and radiation. As scientific research continues to make progress, many drugs have been developed to improve the treatment efficiency for specific types of cancer. Radiotherapy is based on high‐energy radiation to kill cancer cells and shrink tumors.^2^ Normal tissues are relatively insensitive to radiation and are often spared during treatment. Chemotherapy is administered to inhibit the growth of cancer cells, kill cancer cells, or block cancer cell proliferation. However, clinical trials have repeatedly shown no significant increase in survival between some cancer patients who received postoperative chemoradiotherapy and those who only underwent surgery.^3^, ^4^, ^5^

The radiation response of a tumor, which is linked to radiosensitivity and radioresistance, is the key factor in determining the therapeutic effect.^6^ DNA damage induced by radiotherapy eventually influences cell proliferation and changes the cell cycle, leading to apoptosis or other programmed death pathways.^7^ Chemoresistance can be classified as primary drug resistance (PDR) or multidrug resistance (MDR). PDR refers to patients who develop drug resistance only to the inducing drug and do not show cross‐resistance with other drugs, whereas MDR refers to tumor cells that are resistant not only to the original antitumor drug but also to other antitumor drugs with different structures and mechanisms of action^8^; this is the primary cause of chemotherapy failure. More chemotherapy drugs than ever are clinically available for cancer patients including oxaliplatin, Taxol, cisplatin (CDDP), and 5‐fluorouracil (5‐FU). In general, chemotherapy regimens for cancer are administered as monotherapy or two‐drug polytherapy. Different treatment plans can be administered for different conditions to achieve the optimal therapeutic effect. In addition, radiation causes a series of physical and chemical reactions, and cells may lose their ability to divide and die. Therefore, understanding the genes that affect therapeutic resistance is useful for exploring drugs that reverse chemo‐ and radiotherapy resistance.^9^

Two types of genes are involved in the process of cell tumorigenesis: oncogenes (such as epidermal growth factor receptor [EGFR] and human epidermal growth factor receptor 2 [HER2]), which cause normal cells to become cancerous, and tumor suppressor genes (such as p53), which inhibit cell proliferation and tumorigenesis and are often mutated in tumors.^10^ Elucidating the relationship between these genes and chemo‐ and radiotherapy resistance in cancer patients undergoing these treatment modalities may have profound impacts on the treatment and prevention of cancer. This review aims to expand the knowledge on the genetic mechanisms associated with radio‐ and chemoresistance in cancer, which may provide new ideas for the development of targeted therapy.

## 2 MOLECULAR MECHANISMS OF RADIORESISTANCE IN CANCER

### 2.1 DNA damage repair

Radiation therapy is known to either directly induce DNA damage via ionizing radiation (IR) or indirectly promote the absorption of high‐energy wavelengths by other molecules surrounding DNA, resulting in highly reactive free radicals that can damage DNA.^11^ These radicals induce the formation of reactive oxygen species (ROS) and subsequent oxidative stress, and the cancer cells are eventually injured.^12^ To kill tumor cells, radiotherapy causes various forms of damage to intracellular DNA, such as the generation of abasic sites, single‐strand breaks (SSBs) and double‐strand breaks (DSBs) in DNA; DSBs are the most deleterious.^13^ In response, cancer cells activate a series of complex reactions to survive, which can lead to the cancer recurring after DNA damage. However, how do cells sense DNA damage and respond accordingly? Taking DSBs as an example, Rad24p, phosphorylated H2AX (γH2AX), the NBS1/hMRE11/hRAD50 complex, Ku (Ku70/Ku80 heterodimer), mediator of DNA damage checkpoint protein 1 (MDC1), and tumor suppressor p53 binding protein 1 (53BP1) are involved in sensing DNA damage and activating downstream pathways in response. As a DNA damage sensor, Rad24p forms a complex with Ddc1p and Mec3p and induced cell cycle arrest after DNA damage. In addition, the Rad24p‐Rfc2p or Rad24p‐Rfc5p complexes can recruit the Rad24p‐Ddc1p‐Mec3p complex to produce a series of cascade reactions, thereby triggering downstream kinases or effectors such as Rad53p8.^14^ Nonhomologous end joining (NHEJ)‐mediated DSBs are recognized by the Ku protein, which forms an open loop structure connected to the end of the DNA, with one side of the loop forming a scaffold that protects one surface of the DNA double helix, and the other side allowing other NHEJ factors to enter the DSB. Another approach to DNA repair, homologous recombination (HR), includes many mechanisms. HR is always activated in response to SSBs and is promoted by various proteins including the MRE11‐RAD50‐NBS1 (MRN) complex. In addition, the DNA damage signaling system that relies on ataxia telangiectasia mutated (ATM) and ataxia telangiectasia and Rad3‐related (ATR) is closely related to the activation of the HR pathway.^15^, ^16^ DNA damage sensor proteins can not only detect DNA damage but also recruit transducer proteins to provide signals to enzymes to promote DNA repair. There are four main DNA repair pathways in tumor cells: DSB repair, base excision repair (BER), nucleotide excision repair (NER), and mismatch repair (MMR).^17^ For all these processes, multiple sensor proteins are involved such as γH2AX, 53BP1, NBS1, BRCA1/2, and Ku.^14^ There is a direct relationship between MDC1, γH2AX, and 53BP1 expression and DNA breaks: the greater the number of DNA breaks, the higher the level of γH2AX/53BP1 expression is. Therefore, detecting the expression levels of these sensors may serve as predictive biomarkers for determining the outcome of radiotherapy in cancer patients. For example, γH2AX, which can be detected by focal immunocytochemistry or immunofluorescence staining, has been used clinically as a predictive biomarker of radiotherapy sensitivity in certain cancers.^14^ According to the theory of radiobiology, cells with a strong ability to activate DSB repair will develop radioresistance, and cells with weaker repair ability are more sensitive to the killing effect of radiation. In addition, IR activates the expression of phosphatidylinositol‐3 kinases (PIKKs), including ATM, ATR, and DNA‐dependent protein kinases (DNA‐PKs), which have the ability to transform and amplify DNA damage signals.^18^ Finally, the components of DNA repair are recruited to the damaged site and initiate their repair activities.^19^ Thus, when radiation induces DNA damage, the cells can either engage in successful repair promotes to survival or leave the DNA unrepaired and eventually undergo cell death. Hence, regulating tumor cells’ sensitivity to radiation by affecting the DNA damage repair mechanism has been one of the momentous research directions for improving cancer treatment.

#### 2.1.1 X‐ray repair cross‐complementing 1 (XRCC1)

X‐ray repair cross‐complementing 1 (XRCC1) was the first gene found to affect cell sensitivity to IR.^20^ The protein encoded by the XRCC1 gene can efficiently and quickly repair DNA damage due to IR, oxidation, methylation, and other processes, although XRCC1 mutations increase the sensitivity to these damage‐causing effects.^21^ In head and neck squamous cell carcinoma (HNSCC) patients, XRCC1 is associated with poorer survival, especially in patients receiving combined chemoradiotherapy.^22^ Studies have shown that esophageal cancer patients with negative XRCC1 protein expression have slightly higher responses to radiation than those with positive expression, suggesting that patients without XRCC1 protein expression may receive a superior benefit from radiotherapy to some extent.^23^ Labudova et al reported that radioresistant mice receiving high‐dose X‐ray whole body irradiation showed a higher level of XRCC1 gene transcription in the spleen, heart, and kidneys.^24^, ^25^, ^26^ Moreover, XRCC1 is closely related to side effects caused by radiotherapy in breast cancer patients.^27^ In hepatocellular carcinoma (HCC) cells, shRNA‐mediated inhibition of XRCC1 expression could increase DNA damage and cell cycle arrest, thereby making cancer cells more sensitive to γ‐rays; this might be due to decreases in DNA‐PKcs and gadd153 mRNA levels.^28^ In addition, XRCC1 gene polymorphisms are closely related to radiotherapy in various cancers. Three main coding polymorphisms of XRCC1 at codon 194, codon 280, and codon 399 have been identified, which may affect DNA repair ability and subsequently cancer susceptibility.^29^ A study on nasopharyngeal cancer (NPC) found that patients with XRCC1 codon 399 Arg/Arg have a higher risk of developing acute radiation dermatitis.^30^ When evaluating the correlation between the XRCC1 Arg194Trp polymorphism and clinical outcomes in patients with HNSCC undergoing chemoradiation therapy (CCRT), Sambit Swarup Nanda et al reported that the incidence of acute radiation in HNSCC patients with the polymorphic variant XRCC1 who were treated with CCRT increased significantly, and others have stated that genetic polymorphisms in XRCC1 may affect the effect of radiotherapy in prostate cancer, breast cancer, and lung cancer.^24^, ^31^, ^32^, ^33^, ^34^, ^35^ Patients with XRCC1 gene polymorphisms are more likely to experience severe acute dermatitis and oral mucositis, and this information can be used to develop personalized radiation therapy strategies.^36^

#### 2.1.2 Replication protein A (RPA)

Replication protein A (RPA), a single‐strand DNA‐binding protein in eukaryotes, is a trimer consisting of RPA1, RPA2, and RPA3 and plays an indispensable role in DNA replication, damage repair, and cell cycle regulation.^37^, ^38^ The sensitivity of tumor cells to radiation‐induced damage is closely related to their capacity to repair damage, and RPA may be involved in the process of radiation resistance. Ogawa found that the downregulation of RPA gene expression in a highly sensitive esophageal cancer cell line was detected with biochip technology.^39^ Inhibition of RPA gene expression suppresses cellular DNA repair after radiation exposure and is speculated to have an impact on radiosensitization. The sensitivity of radiation‐resistant esophageal cancer cells to radiation can be enhanced by inhibiting RPA1 or RPA2 expression.^40^ In NPC, RPA1 protein is frequently overexpressed, and loss of RPA1 enhances the radiosensitivity of cells.^41^ In glioblastoma (GBM), high RPA expression indicates poor patient survival, and silencing RPA expression impairs the survival and self‐renewal capacity of GBM cancer stem‐like cells (GSCs) and their sensitivity to IR.^42^ Chemical inhibition of RPA with (1Z)‐1‐[(2‐hydroxyanilino)methylidene]naphthalen‐2‐one (HAMNO) induces DSBs, damages the DNA repair ability of GSCs, and ultimately increases their sensitivity to radiation. HAMNO treatment causes DNA replication stress in cancer cells that are ready to undergo replication but not in normal cells, and it works in conjunction with etoposide to kill cancer cells in vitro and suppress tumor growth in vivo.^43^ The underlying mechanism may involve changes in the cell cycle distribution caused by G2/M arrest, which, in turn, reduces the repair of sublethal damage in irradiated cells.^40^ Inhibited RPA negatively mediates the timely repair of damaged DNA, which is a radiation‐sensitive mechanism. Since RPA controls many key proteins and cross‐linked signaling pathways involved in DNA repair, the mechanism by which RPA inhibition induces sensitivity to radiotherapy is the result of the combined action of many factors. However, the use of HAMNO as an RPA inhibitor needs further validation in clinical trials, and its side effects need to be evaluated. Overall, RPA may become a new target for the radiation sensitization of cancer cells.

#### 2.1.3 Poly(ADP‐ribose) polymerases (PARPs)

Poly(ADP‐ribosyl)ation (PARylation) is a post‐translational modification of histones and other nuclear proteins that directly depends on the presence of DNA damage and promotes the survival of damaged proliferating cells.^44^ PAR is synthesized from NAD+ by PARPs,^45^ which consist of 18 proteins encoded by different genes^44^ and play a role in radiotherapy tolerance of cancers. After IR, PARP‐1 promotes autophagy by activating the adenosine monophosphate‐activated protein kinase (AMPK)/mechanistic (or mammalian) target of rapamycin (mTOR) pathway, thereby inhibiting radiation sensitivity in human NPC cells.^46^ PARP inhibitors affect the growth, survival and radiosensitivity of human alveolar and embryonic rhabdomyosarcoma cell lines.^47^ The PARP inhibitor olaparib has been shown to enhance the radiosensitization effect on high‐grade serous ovarian cancer, and inhibition of PARP1 seems to regulate the recombination of DNA strand breaks and affect the concentration of ROS.^48^, ^49^ PARP inhibition also sensitizes small cell lung cancer (SCLC) cell lines and patient‐derived xenografts (PDXs) to IR and may provide a new approach for improving the efficacy of SCLC radiotherapy.^50^ Administration of PARP‐1 inhibitors (rucaparib and olaparib) to patients with high‐risk neuroblastoma can make cancer cells sensitive to X‐rays, and its mechanism of action is likely to be accumulated DNA damage.^51^ However, the following side effects are common in patients treated with olaparib (incidence over 30%): reduced hemoglobin, nausea, and fatigue. In conclusion, PARPs play an important role in tumor radiotherapy resistance by affecting DNA damage repair, autophagy, and apoptosis. The discovery of PARP inhibitors is of great significance in breaking through the problem of radiotherapy resistance.

### 2.2 Cell cycle redistribution

Maintaining the integrity of the genome after DNA damage is essential for the proliferation and survival of eukaryotic cells. Upon activating a series of biochemical reactions in response to DNA damage, the cells undergo apoptosis or become senescent when the damage is too severe or continue to stay in the cell cycle for a longer period of time to enhance the DNA repair mechanism if the damage is not irreversible.^52^ DNA damage causes cell cycle arrest, such as the activation of the G2/M checkpoints triggered by ATM and ATR, which prevent cells from entering mitosis with damaged DNA.^53^ In cells, the MRN complex is recruited in response to DNA damage and promotes the activation of ATM and the recognition of DSBs.^18^

#### 2.2.1 Ataxia telangiectasia mutated (ATM) pathway

Cell cycle checkpoint regulation is one of the signaling pathways important for DNA damage repair that protects cancer cells from radiation‐induced DNA damage.^2^ Cells need time to repair DNA DSBs after they are arrested in the G1/S or G2/M transitions. ATM is a phosphatidylinositol kinase‐related protein that modulates cell cycle checkpoints after DNA damage is induced by IR.^54^ Activation of ATM leads to dimer dissociation, autophosphorylation, and phosphorylation of downstream proteins including p53 and checkpoint kinase 2 (Chk2).^2^ p53 is a crucial cell cycle checkpoint regulatory protein that induces G1/S phase arrest by activating p21. The Chk2 pathway can be used to modulate the G2/M phase transition when the cell division cycle protein 2 (Cdc2)/cyclinB complex is activated.^55^ High expression of ATM was found to increase the efficiency of DNA damage repair and promote radiation resistance, and ATM protein expression correlates with radioresistance in human cancer.^56^, ^57^ Inhibition of ATM expression can enhance the sensitivity of cancer cells to IR by delaying DNA DSB repair, preventing Nuclear factor‐κB (NF‐κB) translocation, inhibiting phosphorylation of p38 and inducing of cJun N terminal kinase (JNK) activity.^58^, ^59^, ^60^ In summary, ATM is overexpressed in a variety of radiation‐resistant tumors, and the ATM pathway regulates cell cycle checkpoints to make cancer cells resistant to radiotherapy after IR‐induced DNA damage. Cell division continues through the cell cycle but is halted by radiation resulting in DNA damage and subsequent cell death; however, some of the damaged cells are able to repair the damage, indicating that ATM inhibition may be a means to overcome radiation resistance.

### 2.3 Epithelial‐mesenchymal transition

Epithelial‐mesenchymal transition (EMT) is the process by which epithelial cells transform into mesenchymal cells and acquire migratory abilities.^61^ Cancer cells undergo EMT, which create a favorable microenvironment for cancer development and metastasis, and the acquisition of EMT functionality is associated with resistance to radiotherapy and poor prognosis in multiple types of malignant tumors. One of the characteristics of EMT is the loss of intercellular adhesion and decreased expression of E‐cadherin; this progressive decline is regulated by the zinc finger proteins Snail and Slug.^62^ Studies have shown that radiation activates the Wnt/β‐catenin pathway and EMT in esophageal squamous cell carcinoma (ESCC) cells and CRC cells.^63^, ^64^ According to previous research on the radiation‐resistant KYSE‐150RR esophageal cancer cell line, the effects of radiation exposure mostly depend on downregulation of phosphatase and tensin homolog (PTEN) expression and activation of the PK B (Akt)/Snail signaling pathway to induce EMT.^65^ Moreover, PTEN, a possible tumor suppressor gene, can enhance cell radiosensitivity by acting on phosphatidylinositol‐3‐kinase (PI3K) to suppress tumor proliferation and metastasis.^66^ In NPC, residual postirradiated cells showed enhanced radioresistance and cross‐resistance to paclitaxel and cisplatin compared to cells before radiation. At the same time, downregulation of E‐cadherin expression and upregulation of vimentin expression were detected in the remaining cells and tissues.^67^ Similarly, non‐small‐cell lung cancer (NSCLC) cells that survived IR therapy also showed an EMT phenotype.^68^ The characteristic changes associated with the EMT phenotype in the tumor microenvironment are related to the resistance of prostate cancer patients to radiotherapy.^69^ In HNSCC tumors, EMT is also associated with the CD44 high/EGFR low phenotype and mitigates the effect of radiotherapy.^70^ Therefore, reversing the metastatic properties of radiation‐resistant esophageal cancer may be an effective strategy for cancer treatment.

### 2.4 Cancer stem cells

**FIGURE 1:**
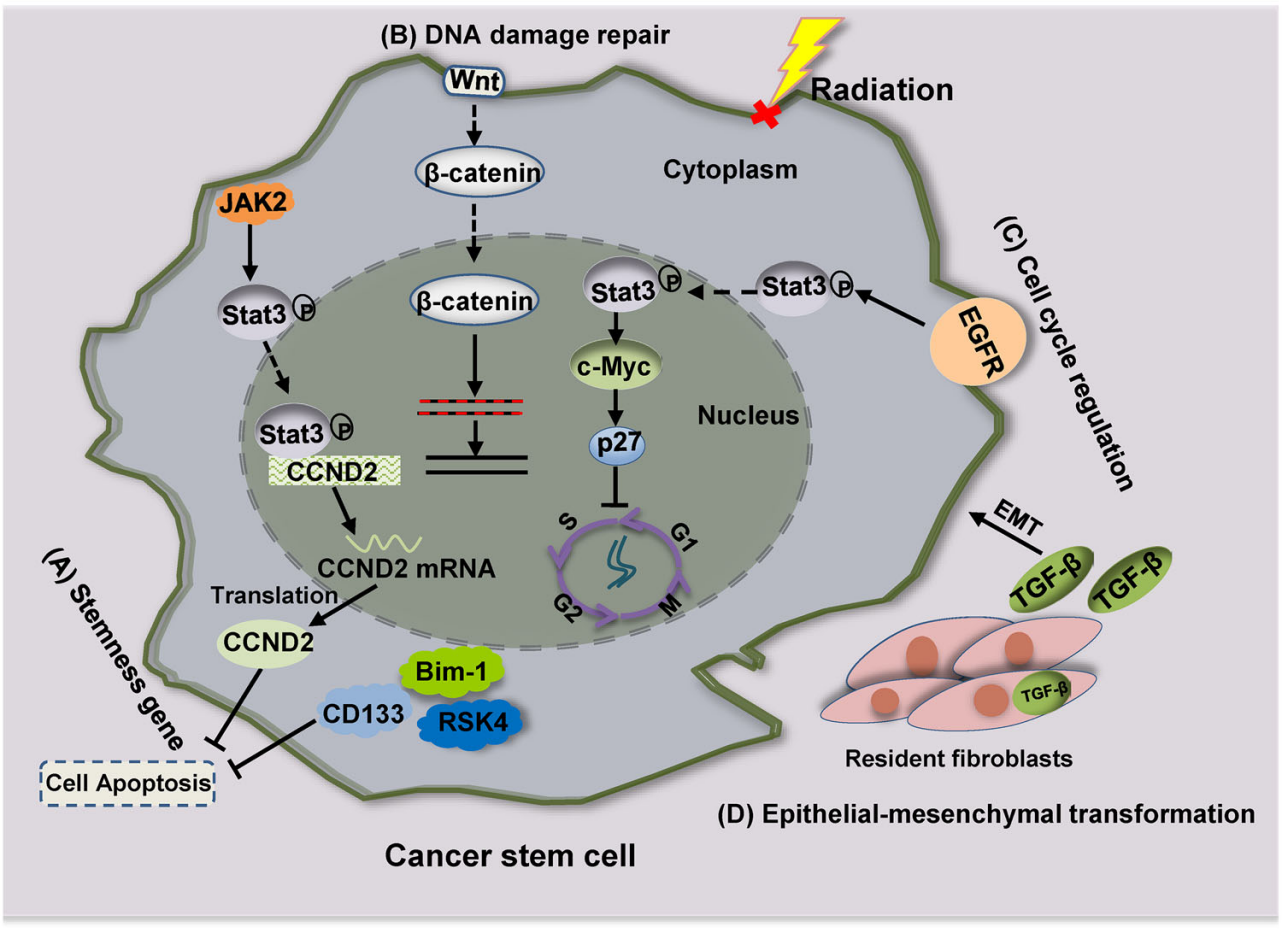
The abnormality of genes contributes to the formation of radiation resistance in CSCs. (A) High expression of Bim‐1, RSK4, CD133, and JAK2 induces cancer cells resistance to radiation. (B) Activation of the Wnt/β‐catenin signaling pathway not only enhances DNA damage repair in CSCs but also promotes the EMT, which induces radioresistance. (C) EGFR/Stat3/c‐Myc/p27 pathway contributes to the cell quiescence and lead to abnormal cell cycle in cancer. (D) TGF‐β secreted by resident fibroblasts promotes the EMT of CSCs, which may decrease radiosensitivity

CSCs are undifferentiated cancer cells with high tumorigenicity, self‐renewal ability, and multidirectional differentiation potential.^71^ Reestablished proliferation of surviving CSCs leads to tumor recurrence and/or distant metastasis because of the radiotherapy resistance of these cells.^72^ Conversely, the generation of CSCs may represent a novel mechanism of resistance to radiotherapy. Various signaling molecules are activated in cancer during radiotherapy, often resulting in irreparable DNA damage and apoptosis. Radiation‐induced DNA damage is generally ineffective because of the high expression of stemness genes, efficient DNA damage repair, and aberrant regulation of the cell cycle, all of which make CSCs less susceptible to radiation (Figure 1).^73^

#### 2.4.1 High expression of stemness genes

Zhang et al obtained a subset of radiation‐resistant esophageal cancer cells by continuous radiation partitioning, which revealed the characteristics of CSCs.^74^ It is possible that stemness genes influence radiotherapy resistance; Janus kinase 2 (JAK2) is overexpressed in CRC cells and is accompanied by the phosphorylation of Signal transducer and activator of transcription (STAT) proteins (especially Stat3). Binding of Stat3 to the cyclin D2 (CCND2) promoter increases the transcription of CCND2 to maintain a complete cell cycle and allow for low‐level DNA damage accumulation, thereby inhibiting apoptosis, enhancing clonal formation, and promoting tumor radioresistance.^75^ The high expression of RSK4 in esophageal cancer stem cells (ECSCs) is related to radiation resistance and poor survival in ESCC patients. The pharmacological inhibition of RSK4 can significantly reduce the characteristics of CSCs and improve radiosensitivity.^76^ As a new CSC target, the combination of The L1 cell adhesion molecule (L1CAM) and CD133 defines a new cancer cell population of ovarian tumor‐initiating cells. L1CAM+/CD133+ cells retain the highest colony formation ability after irradiation and exhibit upregulated expression of some CSC‐specific genes.^77^ CD133+ cells in HCC show cancer‐like characteristics, and silencing CD133 inhibits the growth of liver cancer stem cells (LCSCs) in vivo and in vitro. In addition, inhibiting CD133 can regulate B‐cell lymphoma protein 2 (Bcl‐2) and B‐cell lymphoma protein 2 (Bcl‐2)‐associated X (Bax) to reduce the number of cells in G0/G1 phase, increase apoptosis, and enhance the sensitivity of LCSCs to chemotherapy and radiotherapy.^78^ Bmi‐1, as one of the core members of the polycomb group (PcG) family, not only regulates cell proliferation and differentiation and plays key roles in maintaining the self‐renewal and multidirectional differentiation of normal stem cells but also renews tumor cells into CSCs.^79^, ^80^ Wang et al also verified that Bmi‐1 expression was significantly higher in ECSCs with radioresistance properties than in parental cells, whereas depletion of Bmi‐1 enhanced the radiation response by inducing apoptosis and increasing the levels of ROS, oxidase, and γH2AX.^81^

#### 2.4.2 DNA damage repair and epithelial‐mesenchymal transition

Emerging evidence suggests that high‐efficiency DNA damage detection points and enhanced damage repair ability in CSCs result in their resistance to radiotherapy.^82^ ECSCs have been reported to prevent their apoptosis by incurring reduced DNA damage and exhibiting increased DNA damage repair.^83^ CD133+ lung cancer cells exhibit IR resistance, which is due to enhanced DNA DSB repair in cancer cells and the upregulated expression of DSB repair genes.^84^ Studies have shown that, in lung cancer, Interleukin 6 (IL‐6) can not only promote the self‐renewal of CD133+ CSC‐like cells but also promote DNA repair and protect CD133+ CSC‐like cells from apoptosis and death.^85^ Wnt/β‐catenin pathway activation in response to DNA damage upregulates the downstream signal transduction pathway and prompts efficient DNA repair mechanisms that endow CSCs with higher radiation tolerance.^86^ Therefore, upregulation of Wnt/β‐catenin signaling pathway proteins (Wnt1, Frizzled class receptor (FZD) 1–4, Glycogen synthase kinase‐3beta (GSK3β), CTNNB1 and cyclin D1), increased phosphorylation of GSK3β, and decreased phosphorylation of β‐catenin were also observed in the cells. Transforming growth factor β (TGF‐β)‐induced EMT can regulate radiation tolerance, cell cycle distribution, and free radical scavengers in breast cancer stem cell (CSC‐like cells, all of which seem to be the intrinsic determinants of cell radiosensitivity).^87^ In addition, resident fibroblasts secrete TGF‐β and promote EMT in CSCs, which could decrease the radiosensitivity of these cells.

#### 2.4.3 Aberrant regulation of the cell cycle

Most mature CSCs remain in the quiescent stage, which is a result of their tolerance to radiation. The radiosensitivity of cells differs based on the phase of the cell cycle. As a key mechanism in regulating HR, the suppression of DNA repair protein RAD51 homolog 1 (RAD51) arrests radiation‐resistant GSCs at G2 phase after irradiation.^88^ M phase cells are particularly sensitive, while G0 phase cells are the most resistant.^89^ Stem cells are usually maintained in the G0 phase, a quiescent state in the cell cycle. After radiation exposure, ECSCs can avoid DNA damage caused by radiation because they remain quiescent for a long time.^83^ The proportion of quiescent ECSCs may be higher than that of non‐ECSCs due to increased cyclin D1 and decreased cyclin E levels. Moreover, significantly decreased expression of EGFR, phosphorylated Stat3 and c‐Myc, and significantly increased expression of p27 have been found in ECSCs.^83^ Mechanistically, EGFR activates Stat3, and phosphorylated Stat3 enters the nucleus to promote c‐Myc expression, which inhibits p27.^83^ p27 is a cyclin‐dependent kinase (CDK) inhibitor that restricts cell cycle progression by arresting cells in G1 phase.^90^ Tong et al revealed that low p27 expression may alter the cell cycle to make esophageal cancer cells more resistant to radiation.^91^ This process suggests that the EGFR/Stat3/c‐Myc/p27 pathway may contribute to the quiescence of ECSCs.^83^, ^92^

### 2.5 Multiple signaling pathways promote cell survival and proliferation

DNA damage and the oxidative emergency response caused by irradiation activate specific signaling pathways in cells. Apoptosis or survival signaling pathways may be activated depending on the degree of DNA damage. Studies have shown that the Wnt/β‐catenin pathway, NF‐κB pathway, Akt/cyclin D1/CDK4 survival signaling pathway, and autophagy are associated with radiological resistance in cancer.^86^, ^93^, ^94^, ^95^, ^96^

#### 2.5.1 Autophagy

Autophagy mediates the degradation of dysfunctional organelles and promotes the turnover of long‐lived proteins via a highly conserved process. The activation of the autophagy pathway serves as a survival and adaptive mechanism that provides metabolic support in the presence of cellular stressors (such as exposure to radiation), thus, limiting the efficacy of radiation therapy.^97^ It has been reported that autophagy induced by radiation protects breast cancer cells from radiation damage.^98^ Clinical studies indicate that hypoxia stimulates autophagy and increases radiation resistance in tumor cells.^99^ Mechanistically, hypoxia‐induced autophagy contributes to radioresistance via c‐Jun‐mediated Beclin1 expression in lung cancer cells, and Beclin1 induces autophagy mainly by inhibiting osteopontin.^100^, ^101^ In addition, in NPC, LAPTM4B interacts with EGFR and Beclin1 to promote autophagy, and knocking down LAPTM4B can inhibit autophagy and increase the radiosensitivity of cells.^102^ The effect of the autophagy inhibitor 3‐methyladenine (3‐MA) on radiosensitivity has been tested in the human ESCC cell line. The results showed that radiation can induce the accumulation of autophagosomes, and 3‐MA effectively inhibited this activity. The suppression of autophagy also increased the apoptosis rate and inhibited tumor cell proliferation, which led to radiosensitivity of ESCC cells in vitro and in vivo.^94^ Lys05 has the potential to accumulate in lysosomes and consequently block autophagy, and the combined use of Lys05 and IR can significantly reduce cell survival.^103^ PI3K/Akt/mTOR pathway inhibitors can also reduce autophagy and enhance the radiosensitivity of prostate cancer cells.^104^ Therefore, blocking autophagy may be a reasonable strategy for increasing the sensitivity of esophageal cancer cells to radiotherapy.

#### 2.5.2 Nuclear factor‐κB pathway

Activated NF‐κB signaling regulates its downstream target genes, such as cyclin D1 and c‐Myc, mitigates apoptosis and induces the proliferation, invasion, and metastasis of tumor cells as well as their resistance to radiotherapy and chemotherapy.^105^ In gliomas, activation of the prostaglandin‐endoperoxide synthase 2 (PTGS2)/NF‐κB signaling pathway enhances the radioresistance of cells.^106^ In human HCC, Aurora‐A enhances the activity of NF‐κB and promotes the expression of its downstream effectors, such as Mcl‐1, Bcl‐2, PARP, and caspase‐3, thereby reducing radiation‐induced apoptosis of parental cells.^107^ Similar findings were also observed in breast cancer cells and melanoma cells, indicating the potential of targeting NF‐κB to overcome radiotherapy resistance.^108^, ^109^ Blockade of the NF‐κB signaling pathway can increase the sensitivity of tumors to radiotherapy. In NSCLC, ginsenoside Rg3 inhibits NF‐κB activation, which in turn reduces the expression of NF‐κB gene products and makes lung cancer cells sensitive to gamma radiation.^110^ In colorectal cancer cells, the inhibitory effect of allicin and curcumin on NF‐κB can also increase the sensitivity of cells to radiotherapy.^111^, ^112^ Studies have shown that the inhibitor NS398 can reduce the proliferation of esophageal cancer cells and has a radiosensitizing effect, which is associated with a decrease in NF‐κB activity. Therefore, the radiosensitivity of cancer is closely related to NF‐κB activity, and inhibiting the NF‐κB pathway can increase radiosensitivity.^95^ This association may open up a new strategy for the treatment of esophageal cancer.

**FIGURE 2:**
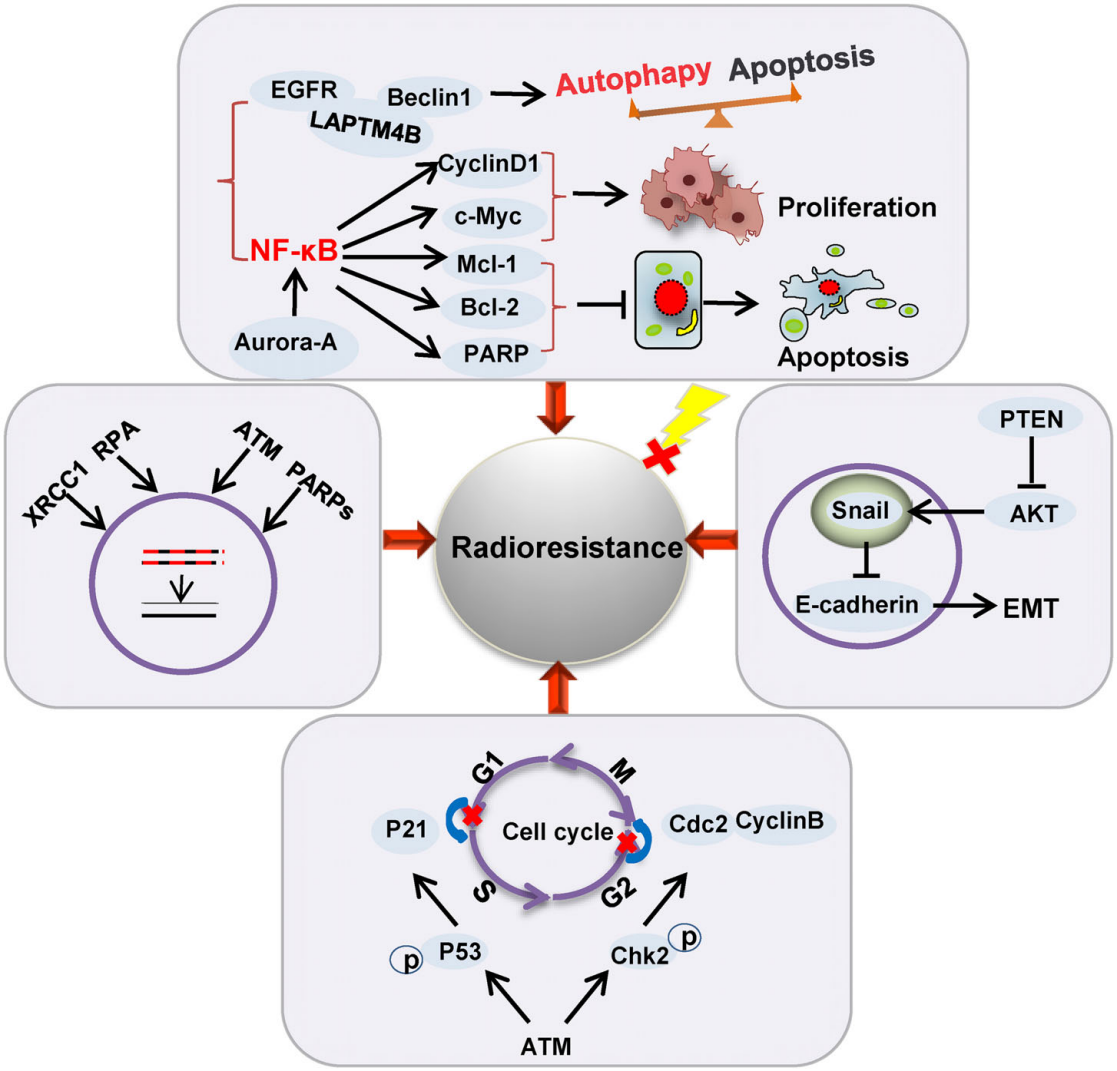
Roles of genes in radioresistant cancer cells. Enhanced DNA damage repair, cell cycle redistribution, EMT, and activation of signaling pathways that promote survival and proliferation contribute to forming radioresistance in cancer

Overall, cancer cells may acquire resistance to radiation by enhancing DNA damage repair, activating cell checkpoint pathways to influence the cell cycle, inducing EMT, and activating a variety of signaling pathways that promote their survival and proliferation (Figure 2). Radioresistance in esophageal cancer is closely related to genetic background, and genetic alterations of the key regulators in cells ultimately affect the therapeutic outcome. Novel therapeutic strategies are also urgently needed to improve radiosensitivity.

## 3 MOLECULAR MECHANISMS OF CHEMORESISTANCE IN CANCER

### 3.1 Cisplatin resistance

Cisplatin, a widely used first‐line chemotherapeutic drug for cancer, is one of the most effective chemotherapeutic agents for various malignancies (e.g., testicular cancer and ovarian cancer).^113^, ^114^ For example, when surgery is combined with cisplatin‐based neoadjuvant chemotherapy, the survival rate of esophageal cancer patients can be significantly improved by 5 years.^115^ However, drug resistance varies widely among cancer patients, and some patients tend to develop chemoresistance to cisplatin and other chemotherapeutic drugs.^116^ Cisplatin cross‐links with DNA to form cisplatin adducts, which subsequently interfere with the basic processes of DNA replication and transcription and ultimately induce apoptosis.^117^ Resistance to cisplatin in the clinical treatment of cancer may lead to treatment failure, and this resistance is determined by a variety of factors. EGFR, a tyrosine kinase receptor, is a major regulator of signaling pathways involved in cell survival, migration, and tissue regeneration.^118^ Clinical studies have shown that among patients treated with neoadjuvant cisplatin, nonreactive patients more frequently have tumors with EGFR overexpression, suggesting that EGFR is a key factor in chemoresistance to cisplatin.^119^ Aside from EGFR overexpression, cisplatin resistance is acquired through several other mechanisms.

#### 3.1.1 Reducing the accumulation of cisplatin within tumor cells

Attenuated drug accumulation is usually observed in cisplatin‐resistant cell lines.^120^ Copper transporter receptor 1 (CTR1) has been suggested to promote the uptake of cisplatin, and the other two copper transporters, p‐type ATPase copper transporting alpha (ATP7A) and ATPase copper transporting beta (ATP7B), have also been found to be related to the export of cisplatin from cells.^121^ In particular, CTR1, the major copper influx transporter, has been shown to play a significant role in platinum resistance. The researchers examined CTR1 mRNA expression levels in 15 patients with stage III/IV ovarian cancer and found a positive correlation between CTR1 mRNA expression levels and the efficacy of platinum drugs in patients.^122^ Studies have found that antioxidant 1 copper chaperone (ATOX1) may affect the accumulation of cisplatin in cells by mitigating the expression level of CTR1 via ubiquitination modification.^123^ Therefore, researchers suggest that the expression of CTR1 protein in tumor cells is closely related to the emergence of cisplatin resistance in tumor patients. The lower the expression of CTR1 in cells is, the lower the accumulation of platinum‐based drugs and the worse the treatment effect in patients.^124^ In cisplatin‐resistant cells, the expression of ATP7A and ATP7B proteins is upregulated, and overexpression of these proteins can promote cisplatin export from cells, thus, resulting in acquired drug resistance.^125^, ^126^, ^127^ In addition, clinical studies have shown that ATP7B expression levels can be used to predict the sensitivity of ovarian and endometrial cancers to cisplatin treatment.^128^, ^129^, ^130^ However, other reports in human ovarian cancer cell lines have stated that cisplatin resistance may be primarily due to reduced drug uptake.^131^ In addition, MDR‐associated protein 2 (MRP2) may be the main protein used to export drugs from cisplatin‐resistant cells, and its expression level may be used to predict the susceptibility of tumor cells to platinum‐based therapies.^131^, ^132^, ^133^, ^134^ Multidrug resistance 1 (MDR1) protein plays a major role in the forced export of cisplatin.^135^ Studies have reported that entinostat may reverse cisplatin resistance via the Src‐Mcl‐1‐MDR1 pathway in ESCC cells, which also illustrates the significance of MDR1 in the cisplatin resistance of ESCC cells.^136^ Overall, a reduction in the accumulation of cisplatin may still depend on increased efflux and/or decreased uptake in different tumor cells.^137^ The CTR1 protein mentioned in this review has been thoroughly confirmed to affect cisplatin uptake, and a reduction in its expression will contribute to the development of cisplatin resistance. Furthermore, decreased expression of proteins that contribute to cisplatin efflux, such as MDR and MRP2, also results in noticeable changes in the functions in some radiation‐resistant tumor cells.

#### 3.1.2 Inactivation of cisplatin

When cisplatin is hydrolyzed in the cytoplasm, it binds to sulfur‐containing molecules, such as glutathione (GSH), metallothionein, and other proteins containing cysteine residues, which confines cisplatin to the cytoplasm and prevents it from entering the nucleus to bind DNA. Therefore, an increase in intracellular sulfur‐containing molecules may lead to cisplatin resistance. Increased GSH expression has been observed in many cisplatin‐resistant tumor models and has been further confirmed in clinical studies.^138^, ^139^, ^140^ Similar to the reaction of GSH, metallothionein can bind to and inactivate cisplatin, and increases in metallothionein expression level are positively correlated with cisplatin resistance in prostate, lung, ovarian, and cervical cancer.^140^, ^141^, ^142^, ^143^

#### 3.1.3 Enhancing DNA damage repair

Cisplatin can damage DNA and eventually cause apoptosis, but efficient DNA damage repair can lead to chemoresistance in cancer cells. The heterodimer formed by excision repair cross‐complementing gene 1 (ERCC1) and xeroderma pigmentosum complementation group F (XPF) is a specific 5′‐bound endonuclease with the ability to recognize DNA damage and resect the 5′‐end, which has an indispensable part in limiting or regulating speed during NER.^144^, ^145^ Studies have shown that the activity of the ERCC1‐XPF complex can reflect the efficiency of NER, and the ERCC1‐XPF complex repairs DNA DSBs via HR, thus, causing cisplatin resistance in lung cancer.^146^, ^147^ ERCC1 overexpression not only causes damaged DNA to stagnate in G/M phase for rapid repair but also increases the clearance of the DNA complex induced by cisplatin, ultimately resulting in cisplatin resistance.^148^ The mRNA or protein expression level of ERCC1 is negatively correlated with the clinical response or survival of patients administered cisplatin therapy, which is manifested in a variety of tumors including bladder cancer, colorectal cancer, stomach cancer, esophageal cancer, head and neck cancer, and NSCLC.^149^ In addition, the XPF expression level is closely related to cisplatin resistance. Kidney cancer cell lines with high XPF expression were more resistant to cisplatin than were other cell lines. Low XPF expression will reduce the DNA damage repair capacity so that cisplatin resistance is weakened and apoptosis rates increase.^150^ Taken together, these data suggest that cancer cells can be sensitized to cisplatin by decreasing the expression level of ERCC1 or XPF.

#### 3.1.4 Epithelial‐mesenchymal transition

EMT is also correlated with the sensitivity of cancer cells to chemotherapeutic drugs. The expression of pivotal BER gene MutY homolog (MUTYH) was found to be significantly downregulated in an esophageal cancer cell line with cisplatin resistance. CDDP‐resistant cells undergo EMT driven by the master regulator Twist, and MUTYH overexpression prominently reduces Twist expression levels and reverses the EMT phenotype.^151^ In addition to affecting transcription, MUTYH is also related to the degradation of Twist.^152^ Overall, activation of EMT mediated by MUTYH downregulation is one of the mechanisms by which ESCC acquires CDDP resistance. Cisplatin resistance was negatively correlated with eukaryotic translation initiation factor 5A2 (EIF5A2) expression in gastric cancer cells. Knockdown of eIF5A2 was associated with upregulated expression of the epithelial markers E‐cadherin and β‐catenin and decreased expression of the mesenchymal markers vimentin and *N*‐cadherin, indicating that eIF5A2 could reverse EMT process and block the effect of cisplatin on EMT‐related markers.^153^

#### 3.1.5 Inactivation of cell death signaling

Multiple prosurvival pathways that affect proliferation and confer antiapoptotic abilities play important roles in the regulation of the tumor cell response to chemotherapy, leading to poor treatment outcomes.^149^ Upon comparison of the sensitivity of tumor cells with normal and defective p53 to cisplatin, the stability and activation of wild‐type p53 was found to be crucial for cisplatin‐induced apoptosis in a large number of in vitro experiments and clinical trials.^154^, ^155^, ^156^ Ovarian cancer patients with normal expression of wild‐type p53 have been reported to be more likely to achieve good cisplatin outcomes than patients with p53 mutations.^157^, ^158^ p53 can upregulate the death receptor Fas/cluster of differentiation 95 (CD95)/apoptosis antigen 1 (APO‐1) axis to promote apoptosis in testicular germ cell tumors.^159^ However, Fas protein expression is decreased in metastatic colon cancer cells with cisplatin resistance that lacked p53 activity. p53‐mediated transcriptional activation of the death receptor Fas/CD95 pathway may be a major factor in inducing cisplatin sensitivity in p53‐positive tumor cells.^160^

A family of EGFR tyrosine kinase proteins encoded by the Erb‐B2 receptor tyrosine kinase 2 (ERBB2) gene is amplified or highly expressed in multiple tumors.^161^, ^162^ The ERBB2 signal is transmitted through a variety of downstream pathways, including the src homology and collagen (SHC)/growth factor receptor‐bound protein 2 (GRB2)/son of sevenless (SOS) and PI3K/Akt1 signaling pathways.^163^ Cyclin dependent kinase inhibitor 1A (CDKN1A) protein expression is upregulated through the PI3K/Akt 1 pathway in cell homeostasis, while overexpression of ERBB2 promotes CDKN1A translocation from the nucleus.^164^, ^165^ Interestingly, both mechanisms may actually induce cisplatin resistance.^166^ This evidence indicates that ERBB2 overexpression induces cisplatin resistance in NSCLC patients.^167^

Intrinsic or acquired cisplatin resistance of tumor cells limits the use of cisplatin in cancer chemotherapy.^168^ In the clinic, a combination of multiple chemotherapy drugs is often used to treat cancer patients, but cancer cells may be resistant to different classes of chemotherapeutic drugs; therefore, it is necessary to have different chemotherapy strategies based on the specific genetic background of the patients.

### 3.2 5‐Fluorouracil resistance

5‐FU, a pyrimidine derivative in which the hydrogen in the fifth position of uracil is replaced with fluorine, is a first‐line chemotherapy agent for esophageal cancer and is commonly used chemotherapy agent for other solid tumors. The treatment outcomes of individuals with esophageal cancer treated with 5‐FU differs; therefore, overcoming drug resistance and improving the efficacy of this class of anticancer drugs have become crucial obstacles in cancer treatment. As a thymidylate synthase (TS) inhibitor, 5‐FU is metabolized into 5‐fluorouracil deoxynucleotide (5F‐dUMP) in the cell, which inhibits deoxythymidylate synthase, preventing the methylation of deoxyuridylate (dUMP) to deoxythymidylate (dTMP), and ultimately affects DNA synthesis.^169^, ^170^ Moreover, 5‐FU can be converted into a 5‐FU nucleoside in vivo, which is incorporated into RNA as a pseudometabolite, to interfere with protein synthesis; therefore, it impacts cells at multiple stages.^171^ Resistance to 5‐FU is a multifactorial event that may be due to changes in transport mechanisms, metabolism, apoptosis, and cell cycle dynamics.^172^ Understanding the underlying mechanisms of 5‐FU resistance in cancer is an essential step to predict and overcome 5‐FU chemoresistance to improve patient survival.

#### 3.2.1 Antiapoptosis

Apoptosis is a mechanism of cell death commonly induced by chemotherapy, and the failure to initiate apoptosis represents a significant characteristic of chemoresistant tumor cells. As a member of the tryptophan‐aspartic acid repeat protein (WD40) family, receptor for activated C kinase 1 (RACK1) is a necessary participant in transcription and translation events and regulates binding protein activity.^173^ In a recent study, RACK1 was identified as an oncogene in ESCC that promoted cell proliferation.^174^ Overexpression of RACK1 could promote 5‐FU chemoresistance, while downregulation of RACK1 enhanced cell sensitivity to induce apoptosis.^175^ Moreover, RACK1 was found to modulate the activity of Akt, and phosphorylated Akt can increase the expression of Bcl‐2), which is an antiapoptotic member of the Bcl‐2 family.^175^, ^176^, ^177^, ^178^, ^179^ The balance between Bcl‐2 family proteins is vital to the regulation of apoptotic pathways, and Bim is a proapoptotic protein member of Bcl‐2 family.^180^ In esophageal cancer, overexpression of RACK1 was reported to promote the expression of Bcl‐2 and inhibit Bim expression, raising serious questions about resistance to 5‐FU and cisplatin.^175^ Mechanistically, RACK1 has a suppressive effect on apoptosis because it interacts with different partners. Therefore, PI3K/Akt and Bcl‐2 activation induced by RACK1 overexpression may be effective anticancer therapeutic targets for patients with chemoresistant ESCC.^175^, ^181^ Targeting RACK1 may improve the efficacy of ESCC chemotherapy.

STAT‐3 is another transcription factor that is critical for cancer progression and chemoresistance.^182^ Activated Stat3 regulates the transcription of genes that control cell survival, proliferation, and the immune response as well those that are involved in the antiapoptosis response. Stat3 is reported to be activated in many cancers and to influence patient survival.^183^ For example, tyrosine phosphorylation of Stat3 may affect GBM survival^183^ and sensitize colorectal cancer to chemoradiotherapy in vitro and in vivo.^184^ Furthermore, inhibition of Stat3 decreased the expression of cyclin D1 to increase apoptosis in cells treated with 5‐FU.^185^ Exosomal transfer of p‐Stat3 induces 5‐FU resistance in colorectal cancer cells by mitigating activation of the caspase cascade of apoptosis.^186^ Thus, targeting Stat3 may improve the efficacy of cancer chemotherapy.

#### 3.2.2 Thymidylate synthase inhibition

TS is a key enzyme in the metabolism of folic acid and a target enzyme of 5‐FU.^187^ It can promote the conversion of intracellular dUMP to dTMP, which is the only source of new thymidylic acid in cells. Genetic variations in TS functions can alter the toxicity and efficacy of 5‐FU, as indicated by a significant correlation between TS expression and survival of esophageal cancer patients.^188^ Low mRNA expression of TS may indicate a strong response to chemotherapy and longer survival than those of patients with high TS mRNA expression.^188^, ^189^, ^190^ In vitro studies have shown that 5‐FU resistance is related to increased TS activity, and the TS gene has an E2F binding site in its promoter region. Another study identified a detailed E2F transcription factor 1 (E2F1)‐dependent mechanism by which inhibitor of DNA binding 1 (Id1) increases the expression of TS and insulin‐like growth factor 2 (IGF2) to promote esophageal cancer chemoresistance.^191^ Furthermore, oligodeoxynucleotides (ODNs) induced by E2F‐1 overexpression restored the sensitivity of 5‐FU‐resistant DLD‐1 cells to 5‐FU, confirming the importance of E2F‐1 in 5‐FU resistance.^192^ Based on the relationship between TS expression and 5‐FU sensitivity in esophageal cancer, a more effective and individualized therapeutic approach should be established for patients.

#### 3.2.3 Changing the activity of dihydropyrimidine dehydrogenase

Approximately 85% of 5‐FU is inactivated by dihydropyrimidine dehydrogenase (DPD).^193^, ^194^ DPD converts 5‐FU to dihydrofluorouracil, which is then catalyzed by dihydropyrimidinase and β‐ureidopropionase into 5‐fluoro‐ureido‐propionic acid (FUPA) and ultimately removed from the body in urine. Some results show that high DPD gene (DPYD) expression affects 5‐FU resistance, and the expression level of DPYD may be a biomarker for predicting outcomes of 5‐FU‐based chemoradiotherapy.^195^ In a nude mouse model of 5‐FU chemoresistance, DPYD expression is upregulated not only in colon tumor tissues but also in liver, which can accelerate the metabolism of 5‐FU.^196^ The copy number of DPYD genes in the 5‐FU‐resistant TE‐5R esophageal cancer cell line was amplified compared to that in TE‐5 cells, and the mRNA and protein expression levels of DPYD in 5‐FU‐resistant cells was higher than that in wild‐type cells. The concentration of 5‐FU in 5‐FU‐resistant cells was significantly decreased compared with that in the parent cells after 5‐FU treatment, and the concentration of the 5‐FU metabolite FUPA was increased.^197^ More interestingly, the DPYD inhibitor gimeracil distinctly enhanced the intracellular 5‐FU concentration, suppressed the intracellular FUPA concentration, and reduced 5‐FU resistance. These results indicate that one mechanism of 5‐FU resistance is caused by the rapid degradation of 5‐FU due to overexpression of DPYD.^197^ The study of DPYD gene copy number amplification and corresponding DPYD overexpression may provide a new biological basis for exploring prevention and treatment strategies for 5‐FU‐resistant cancer cells.

### 3.3 Taxol resistance

At one point, cisplatin combined with 5‐FU was the standard chemotherapy treatment prescribed for esophageal cancer, but treatment outcomes were unsatisfactory. Taxol (common name paclitaxel) mainly binds to tubulin to block the depolymerization and recycling of microtubules, which activates the checkpoint function during mitosis to arrest cells at metaphase and induce apoptosis.^198^ Recent studies have demonstrated that Taxol is effective in the treatment of advanced breast cancer and ovarian cancer and may lead to outstanding positive effects in cases with adriamycin and cisplatin resistance.^199^ Taxol combined with cisplatin or 5‐FU significantly prolonged the survival of cancer patients in some clinical trials.^200^, ^201^ However, one of the main reasons for the limited application of Taxol is acquired drug resistance. Improving the therapeutic effect and enhancing Taxol‐mediated cell death by apoptosis (or other forms of programmed cell death) has been a hot topic in cancer research.

#### 3.3.1 Cell cycle regulation

Although the pharmacological mechanism of Taxol is complex, its primary effect can be observed in the mitotic stage.^202^ Benzimidazole‐related 1 (BUBR1) is a mitotic checkpoint protein, and dysfunction in its expression and activity can seriously affect checkpoint function.^203^, ^204^ BUBR1 expression in Taxol‐resistant ovarian carcinoma cells is evidently lower than that in parental cells. Furthermore, a weakened spindle checkpoint with decreased expression of BubR1 but not of mitotic arrest deficient 2 (Mad2) promoted acquired Taxol resistance in ovarian carcinoma cells, and regulatory subunit associated protein 2 (CDK5RAP2) was found to regulate the transcription of key mitotic genes, such as BUBR1 and Mad2, to influence resistance to Taxol. Downregulation of CDK5RAP2 expression makes cells insensitive to Taxol, and restoration of CDK5RAP2 expression rescues sensitivity, suggesting that CDK5RAP2 is a target protein of Taxol resistance.^205^, ^206^

#### 3.3.2 Cancer stem cells and antiapoptosis effects

Forkhead box M1 (FoxM1) is a member of the forkhead transcription factor family, a group of proteins that have DNA‐binding domains and can form wing‐like helical structures. FoxM1, which modulates the expression of downstream pathways such as the PI3K‐Akt and RAF‐mitogen‐activated PK (MEK)‐extracellular‐signal‐regulated kinase (ERK) signaling pathways, plays a key regulatory role in cell cycle progression, DNA damage repair and drug resistance.^207^ Recent studies have shown that FoxM1 overexpression promotes the resistance of breast cancer cells to cisplatin, trastuzumab and Taxol.^208^, ^209^, ^210^ FoxM1 regulates prohibitin 1 (PHB1) at the transcription and translation levels, which promotes activation of RAF‐MEK‐ERK signaling, leading to the phosphorylation of ERK1/2 to support the continuous proliferation of cancer cells. On the other hand, FoxM1 positively promotes ATP binding cassette subfamily a member 2 (ABCA2) expression but is also regulated by the FoxM1/PHB1/RAF‐MEK‐ERK feedback loop, which may induce additional resistance of cancer cells to Taxol. Taxol in combination with the FoxM1 inhibitor thiostrepton reversed Taxol resistance and enhanced apoptosis.^211^

Drug resistance is one of the characteristics of CSCs. The Taxol‐resistant ESCC cell line has multiple characteristics of CSCs, which suggests that CSCs may be one of the key mechanisms of Taxol resistance in ESCC.^212^ CSCs are thought to be critical not only for tumorigenesis and cancer maintenance but also for cumulative resistance to chemotherapy and radiotherapy; this resistance manifests due to slowed cell cycle progression, the efflux of drugs by ATP‐binding cassette (ABC) transporters and the upregulated expression of antiapoptosis factors.^213^ The expression level of EIF5A2 is related to poor survival in ESCC patients treated with Taxol after esophagectomy, and the expression levels of multiple transporter genes related to drug resistance were found to be increased in cells overexpressing EIF5A2, suggesting that EIF5A2 overexpression could induce CSC‐specific actions that enhance the chemoresistance of ESCC cells.^214^ Moreover, EIF5A2 can also confer Taxol resistance to ESCC cells by inhibiting apoptosis. N1‐guanyl‐1,7‐diaminoheptane (GC7) has the ability to inhibit EIF5A2 activation; therefore, GC7 may be considered as part of a combination therapy to enhances the sensitivity of ESCC patients to chemotherapy.^215^ High HER2 expression can induce Taxol resistance in breast cancer cells. Drug‐resistant breast cancer cells with high HER2 expression have higher microsphere formation and stem cell markers than CSCs, which are the main contributors to chemotherapy resistance.^216^, ^217^

#### 3.3.3 Epithelial‐mesenchymal transition

Comparison of the differential gene expression patterns between Taxol‐resistant and Taxol‐sensitive breast cancer cells revealed upregulation of EGF‐like repetition and disoidini‐like domain protein 3 (EDIL3),^218^ which encodes an extracellular matrix protein and has been identified as a new regulator of EMT. Knockout of the EDIL3 gene inhibits EMT and sensitizes cells to Taxol; by contrast, overexpression of EDIL3 was reported to induce EMT and Taxol resistance through its interaction with integrin αVβ3. Moreover, EDIL3 may be involved in EMT and Taxol resistance in cancer cells through autocrine or paracrine signaling. Cilengitide blocks the EDIL3‐integrin αVβ3 interaction to restore sensitivity to Taxol and mitigates EMT in Taxol‐resistant cancer cells.^218^ Exploring the genetic basis of Taxol resistance in cancer cells is expected to provide theoretical evidence for a better understanding of the drug response in esophageal cancer.

### 3.4 Oxaliplatin resistance

Oxaliplatin (OXA) is a new generation of platinum‐based antitumor drugs. When OXA enters the cell nucleus, it binds to DNA and forms a variety of cross‐linked structures, resulting in disrupted gene replication and transcription.^219^ Currently, OXA is widely used in the clinic in combination chemotherapies, mainly for malignant tumors of the digestive system. Unfortunately, an increasing number of digestive tract tumors showed OXA resistance, which is the main reason for treatment failure of OXA‐based regimens. Multiple studies have shown that tumor cells must initiate a series of repair measures to survive, and some cells that survive initial drug treatment can transform into dominant resistant cells. In summary, when the drug resistance mechanism of OXA is more comprehensively revealed, its clinical application and therapeutic efficiency will be greatly improved to benefit more cancer patients.

#### 3.4.1 Reduction in intracellular drug concentrations

Numerous reports have found that multiple membrane transporters are highly expressed in OXA‐tolerant colorectal cancer patients, suggesting that membrane transporters promote the occurrence of OXA resistance by reducing drug absorption.^220^ Human copper transporter 1 (hCTR1) exerts its effect by forming a trimer structure and then constructing a tapered pore structure in the cell membrane. Wang et al found that the expression level of hCTR1 was closely related to OXA sensitivity in cells, and increased hCTR1 promoted OXA‐induced apoptosis.^221^ By contrast, decreased hCTR1 expression can reduce apoptosis and enhance drug resistance, for example, OXA exhibits limited therapeutic efficacy when hCTR1 is degraded in HCC.^222^

#### 3.4.2 Destruction of the balance between cell proliferation and apoptosis

Dual specificity tyrosine‐(Y)‐phosphorylation regulated kinases (DYRKs) are involved in the occurrence and development of tumors by regulating cell cycle progression and apoptosis.^223^ Studies have reported that DYRK2 can inhibit tumor cell proliferation via interactions with a variety of tumor suppressor genes, and reduced DYRK2 expression is strongly linked to weak chemotherapeutic efficacy and poor prognosis.^224^ Recently, Zhang et al analyzed the expression of DYRK2 in paired HCC and adjacent tissues from 86 patients and found that the expression of DYRK2 was more significantly downregulated in patients with a higher degree of malignancy and lower survival than in patients with a lower degree of malignancy and better survival.^222^ miR‐141‐3p is significantly increased in ESCC, and its expression level is relevant to the degree of differentiation and tumor‐node‐metastasis (TNM) stage.^225^ An inverse correlation was observed between the expression of PTEN and miR‐141‐3p, and miR‐141‐3p may contribute to the acquisition of chemoresistance in esophageal cancer cells by inhibiting the PTEN expression level to reduce apoptosis in vitro and in vivo, suggesting that the inhibition of miR‐141‐3p may reverse the occurrence of OXA resistance in esophageal cancer.^225^

#### 3.4.3 Cancer stem cells

HCC cell resistance to chemotherapy drugs is closely related to the characteristics of stem cells during treatment based on experiments with OXA as an antitumor drug. Injection of the CSCs generated by OXA treatment into the livers of mice resulted in the reduced sensitivity of liver cells to OXA, and this effect may be related to the secretion of insulin‐like growth factor 1 (IGF‐1).^226^

#### 3.4.4 Enhanced autophagy

Studies have shown that OXA can activate autophagy to produce a significant number of autophagosomes in HCC, thus, promoting drug resistance.^227^ Ren et al showed that miR‐125b participates in the regulation of autophagy through transmembrane protein 166, thereby affecting HCC cell resistance to OXA.^228^ To some extent, the application of specific autophagy inhibitors may improve the efficacy of OXA and inhibit the occurrence of drug resistance to OXA.

#### 3.4.5 Increased DNA repair

Platinum‐based compounds, including OXA, have also been reported to induce the production of free radicals, leading to oxidative DNA damage.^228^ NER uses damage recognition to excise and repair whole‐genome nucleotides.^229^ A mechanism of OXA resistance is mediated by overexpression of the DNA repair protein ERCC1, which is one of the core proteins in the NER pathway. Triptolide can enhance apoptosis and make cancer cells sensitive to DNA damage by inhibiting the DNA damage repair pathway induced by OXA treatment.^229^

### 3.5 Multidrug resistance

**FIGURE 3:**
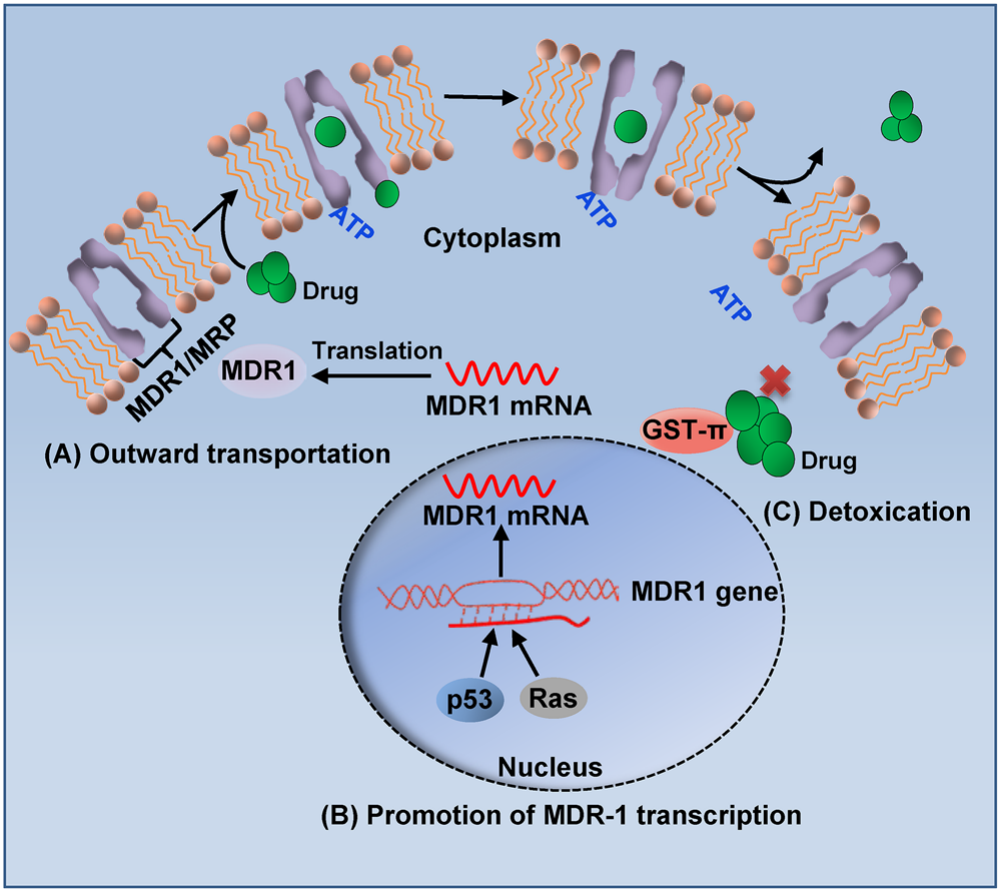
The mechanisms of multidrug resistance. (A) High transport capacity of MDR1 and MRP results in MDR. (B) The p53 and Ras upregulate the transiption MDR1 to increase drug excretion in cancer cells. (C) GST‐π activation of the detoxification system leads to multidrug resistance in cancer cells

Currently, MDR during chemotherapy is believed to be the main reason for the limited clinical efficacy of chemotherapy. Tumor cells resist drugs through a variety of mechanisms, including reduced drug absorption, increased drug efflux, activation of detoxification systems and the DNA repair response, and prevention of drug‐induced apoptosis (Figure 3).^230^ The progress of research on MDR related to esophageal cancer is presented in this section.

#### 3.5.1 Enhancing the capacity of transport proteins

Many membrane proteins, including ABC transporters, play key roles in the efflux of chemotherapeutic drugs, which leads to drug resistance because of a decrease in the effective concentration. High transport capacity is attributed to increased expression of MDR‐associated protein (MRP), P‐glycoprotein 1 (P‐gp) encoded by the MDR1 gene (also known as ABCB1), low‐density lipoprotein receptor‐related protein (LRP), and breast cancer resistance protein (BCRP) in cancer.^231^ At present, it is known that MDR leads to multidrug‐resistant malignant tumors such as leukemia, multiple myeloma, gastric cancer, esophageal cancer, lung cancer, breast cancer, and colorectal cancer. In recent years, many studies have suggested that the MDR1 and MRP genes are highly expressed in advanced tumor cells, which is caused by changes in the expression and amplification ability of the genes during the development of disease.^232^ Eid et al showed that patients with advanced testicular cancer have high expression of MDR1 and poor prognosis, suggesting that MDR1 not only mediates drug resistance but is also related to the malignant biological phenotypes.^233^ Approximately 80% of the MDR1 and MRP genes are expressed in lung cancer naïve to chemotherapy and in 10% in cases after chemotherapy treatment, which indicates that there is indeed increased expression of endogenous and acquired MDR1 and MRP genes in cancer cells. These data suggest that overexpression of MDR1 and MRP genes has some intrinsic connection with MDR and the malignant behaviors of tumor cells.^234^ Moreover, the expression level of P‐gp in cancer tissues remains relatively low after disease progression and could be used to predict drug response during treatment.

#### 3.5.2 Enhanced detoxification of glutathione S transferases (GSTs)

GSTs are essential enzymes for catalyzing glutathione binding reactions and eliminating exogenous toxins from cells. The GSTs associated with tumor tissues include GST‐α, GST‐μ, and GST‐π. GST‐π is one of the most relevant isoforms related to chemoresistance, as indicated by the increased expression of GST‐π in drug‐resistant esophageal cancer cells.^190^, ^235^ Currently, the mechanism by which GST‐π promotes MDR of esophageal cancer cells is thought to include the following aspects. First, GST‐π catalyzes glutathione, which is combined with different negatively charged organic substances to form sulfhydryl compounds, inactivating the drug. Second, GST‐π protein catalyzes nitrosourea antitumor drugs to remove the nitro group and dampen their pharmacological effect. Finally, GST‐π protein eliminates the oxides produced by adriamycin in the human body and promotes its sponging of cellular toxins to protect the cells.^190^

#### 3.5.3 p53 and Ras

Mutations in both oncogenes and tumor suppressor genes have been reported to result in MDR in some cancers. Recent studies have reported that MDR1 expression is correlated with the expression of mutant p53 in well‐differentiated prostate cancer tissues, and its expression in poorly differentiated prostate cancer tissues is high.^236^, ^237^ Mechanistically, investigation into the regulation of the p53 gene on the promoter of the MDR1 gene suggested that mutant p53 protein significantly induces transcriptional activation and expression of the MDR1 gene, thus, enhancing drug resistance.^238^ Ras, a vital oncogene, plays a role in signaling to promote cell growth and differentiation.^239^ The function of Ras protein activation in MDR in multiple cancers has been demonstrated, although the mechanism varies. First, Ras protein can promote drug resistance in cancer by enhancing the protein expression of MDR1.^238^ Second, activated Ras protein can facilitate cell proliferation and inhibit apoptosis via signal transduction, and its expression is positively correlated with treatment response.^240^, ^241^ Third, activated Ras protein can increase GST to enhance its detoxification activity and cause MDR in cancer.^242^ It was found that mutated p53 and Ras genes could significantly activate the promoter of the MDR1 gene in drug‐resistant NH3HT3 cells.^238^ Therefore, the oncogene Ras and tumor suppressor gene p53 are auxiliary players in the MDR of tumors.

MDR in cancer is a complex process affected by many factors. Combining chemotherapeutic drugs and MDR‐reversal agents for cancer treatment may be the future overcoming MDR. Some of the vital genes and signaling pathways involved in cancer chemoresistance are summarized in Table 1.

**TABLE 1:** Genes associated with chemotherapy resistance in cancer

Drugs	Comments	References
Cisplatin resistance	MDR1, GSH, MUTYH, MRP2, CTR1, ATOX1, ERCC1, XPF, EIF5A2, p53, Fas/CD95/APO‐1, CDKN1A, ERBB2	^121^, ^123^, ^133^, ^134^, ^140^, ^151^, ^153^, ^159^, ^162^, ^167^
5‐FU resistance	STAT3, E2F1, DPYD, TS, Id1,DPD	^186^, ^190^, ^191^, ^192^, ^193^, ^196^
Taxol resistance	BubR1, CDK5RAP2, FoxM1, EIF5A2, HER2, EDIL3	^205^, ^206^, ^208^, ^215^, ^216^, ^217^, ^218^
Oxaliplatin resistance	hCTR1, DYRK2, miR‐125b, ERCC1	^221^, ^223^, ^228^, ^229^
Multidrug resistance	MRP, MDR1, GST‐π, Mutation of p53, Ras	^233^, ^234^

## 4 COMBINATION THERAPY

With an increasing number of new drug classes discovered in recent years, targeted molecular therapy has attracted attention for its use in the comprehensive treatment of cancer due to high efficacy and few side effects. Therefore, treatments that target certain genes that are related to radiotherapy and chemotherapy resistance, such as EGFR, HER2, and p53, may be an effective strategy to improve the survival of cancer patients.

### 4.1 Epidermal growth factor receptor inhibitors

EGFR amplification and overexpression is one of the main factors driving cancer development, indicating that EGFR overexpression is related to the generation of resistance to radiotherapy and chemotherapy.^243^ Moreover, anti‐EGFR monoclonal antibodies (cetuximab and panizumab) and tyrosine kinase inhibitors (TKIs) (gefitinib and erlotinib) have conferred significant benefits to participants in clinical trials. This outcome leads to more choices for the treatment of drug‐resistant cancer.^244^ The most commonly mutated gene in NSCLC treated with targeted drugs is EGFR.^245^ Although the first‐ and second‐generation targeted drugs have significant efficacy, two‐thirds of patients develop drug resistance within 1‐2 years after using drugs, and the tumor may recur.^246^ The causes of targeted drug resistance vary among individuals, but 50‐60% of EGFR inhibitor resistance is related to the T790M mutation.^247^ AZD9291 (osimertinib) is an oral third‐generation EGFR‐TKI and the first drug to target EGFR gene mutations (including mutations at residues 18, 19, and 21) and EGFR‐TKI acquired resistance (T790M) in NSCLC.^248^ EAI045, a novel targeted drug that overcomes AZD9291 resistance, can be used in patients with the T790M mutation or the C797S mutation.^249^ If successful in phase III clinical trials, it will usher in the fourth generation of targeted EGFR‐TKIs. In addition, 57 esophageal cancer patients were treated with the combination of cetuximab, paclitaxel, and radiotherapy at a dose of 50.4 Gy/cfx, and the results showed that 70% of these patients had a complete clinical response after radiotherapy and chemotherapy.^250^

### 4.2 Human epidermal growth factor receptor 2 inhibitors

HER2, also known as ErbB‐2, belongs to the EGFR family and has intrinsic tyrosine kinase activity.^251^ HER2 is highly expressed in a variety of tumors, and HER2 overexpression can promote tumor growth, metastasis, and angiogenesis.^252^ HER2 is overexpressed in the tumors of 15‐20% of breast cancer patients, and trastuzumab, a monoclonal antibody targeting HER2, plays a role in the treatment of both early and advanced breast cancer.^253^ However, trastuzumab led to a mild risk of cardiotoxicity usually accompanied by a decline in the asymptomatic left ventricular ejection fraction (LVEF); this sometimes manifested as clinical heart failure.^254^, ^255^ Following trastuzumab, three HER2‐targeted drugs were developed to treat HER2‐overexpressing breast cancer: lapatinib, a small‐molecule dual tyrosine kinase inhibitor that targets EGFR and HER2; trastuzumab DM1 conjugate (also known as T‐DM1), an antibody‐drug conjugate consisting of trastuzumab, a thioether ligand, and derivatives of an antimitogenic drug (maytansine); and pertuzumab, a monoclonal antibody that binds to subdomain II of the HER2 extracellular domain, thereby preventing HER2 from homologous or heterologous dimerization with other HER family receptors.^256^, ^257^, ^258^ Although there are few data on pertuzumab and T‐DM1, the available data indicated that both drugs have lower cardiotoxicity than that of trastuzumab.^259^

Recent results demonstrated that pyrotinib, an irreversible pan‐ERBB2 inhibitor, increased DNA damage and enhanced the radiosensitivity of HER2‐overexpressing gastric and breast cancer cells in vitro and in vivo. Therefore, pyrotinib is a promising irradiation sensitizer in gastric and breast cancer patients with HER2 overexpression.^260^ Pyrotinib also enhanced the cytotoxicity of docetaxel, which may provide a new strategy for potential drug combinations. Cheng reported that high HER2 expression enhances the radiation tolerance of patients with esophageal cancer,^261^ and the anti‐HER2 monoclonal antibody trastuzumab can prolong the survival of patients with HER2‐positive metastatic esophageal cancer.^2^ In a phase III trial of 584 patients with HER2‐positive esophageal cancer, the median overall survival after trastuzumab treatment combined with chemoradiotherapy was longer than that of the patients who received only chemoradiotherapy.^2^

Therefore, investigation into the mechanisms of chemoradiotherapy resistance may provide new ideas for useful therapeutic targets and effective biomarkers, improving the development of targeted drugs that extend the survival of patients with cancer. By exploring the genetic basis of resistance to radiotherapy and chemotherapy in cancer, some theoretical basis for the treatment of other malignant tumors may also emerge.

### 4.3 Other target combinations

A number of mechanisms (cell cycle regulation, DNA damage repair, EMT, CSC resilience, etc.) are all involved in the control of the sensitivity of cancer to radiotherapy and chemotherapy to some extent. Various factors and molecules coordinate with each other to exert an influence on the resistance of cancer cells to radiotherapy and chemotherapy. Modulation of the DNA damage repair response has an important impact on radiotherapy resistance, and radiotherapy sensitization by targeting the relevant resistance genes is beneficial to the clinical treatment of cancer. For example, drugs that reverse EMT, which induces resistance to therapy, may be useful for clinical treatment. Curcumin has been shown to inhibit EMT in drug‐resistant lung cancer by inhibiting the Wnt/β‐catenin pathway, thereby downregulating the expression of the downstream transcription factor Snail.^262^ Therefore, radiotherapy combined with curcumin may contribute to improved cancer treatment.

Cyclin D1 is frequently overexpressed in human cancers and has been reported to be a carcinogenic driver in most of these cancers,^263^ and CDK inhibitors targeting cyclin D1 are considered a feasible method for cancer treatment. Several CDK4/6‐specific inhibitors, including PD‐0332991 (palbociclib), LY2835219 (abemaciclib), and LEE011 (ribociclib), have actually been investigated in depth in preclinical studies and clinical trials.^263^, ^264^, ^265^, ^266^, ^267^ The loss of Rb expression enhances resistance to CDK4/6 inhibitors but increases the sensitivity of tumor cells to combination therapy with CB‐839 and metformin.^268^, ^269^, ^270^ The upregulation of the rate‐limiting enzyme GLS1 in glutamine metabolism directly enhances palbociclib resistance and glutamine addiction. In brief, exploiting relevant factors that can induce resistance may provide new ideas for the clinical treatment of esophageal cancer.

## 5 CONCLUSIONS

This review focuses on the genetic basis of resistance to radiotherapy and chemotherapy in cancer, and the rationale of increasing cell sensitivity by targeting related key genes and signaling pathways has been discussed for use in the clinic. Radiation ionizes molecules and atoms to directly destroy DNA in cells in human tissue. Enhanced DNA repair, cell cycle redistribution, CSC resilience, EMT, and activation of prosurvival pathways are the main mechanisms by which radioresistance is induced in cancer. Many factors, such as ATM, p53, PARP, XRCC1, and Bim‐1, greatly influence cancer radioresistance through these different mechanisms (Table 2). Furthermore, this review describes the role of some genes in the development of resistance to four commonly used chemotherapy drugs: cisplatin, 5‐FU, OXA, and Taxol. Combining targeted drugs with commonly used chemotherapeutic drugs or radiotherapy will prolong the survival of patients with cancer. Cancer cells can develop resistance to chemotherapy through a variety of mechanisms; for example, they can attenuate the accumulation of anticancer drugs, activate detoxification systems, enhance DNA damage repair, and evade drug‐induced cell death.^172^ The phenomenon of MDR during chemotherapy is the main reason for the limited clinical efficiency of chemotherapy. The mechanism of P‐gp drug efflux has been thoroughly studied in MDR.^271^ In summary, cancer cells are able to reduce their sensitivity to radiotherapy and chemotherapy through a variety of methods, thus, contributing to cancer recurrence and treatment failure.

**TABLE 2:** Genes associated with radiotherapy resistance in cancer

Mechanisms	Comments	References
DNA damage repair	ATM, XRCC1, RPA, PARPs, Rad24p, γH2AX, MDC1, 53BP1, NBS1/hMRE11/hRAD50 complex, Ku (Ku70/Ku80 heterodimer)	^14^, ^15^, ^16^, ^20^, ^37^, ^38^, ^44^
Cell cycle redistribution	ATM, p53, p21, Chk2, Cdc2, cyclin B,	^2^, ^54^, ^55^
EMT	PTEN, Akt/Snail, PI3K	^65^, ^66^
CSCs	Bmi‐1, Wnt/β‐catenin, TGF‐β, EGFR/Stat3/c‐Myc/p27, JAK2, RSK4, CD133, RAD51	^75^, ^76^, ^77^, ^78^, ^79^, ^80^, ^81^, ^83^, ^86^, ^87^, ^88^, ^92^
Multiple signaling pathways	NF‐κB pathway, Autophagy, Akt/cyclinD1/CDK4	^97^, ^104^, ^105^

In addition to the aforementioned cancer genes that affect resistance to radiotherapy and chemotherapy, we also discovered that some genes influence not only radiotherapy resistance but also chemotherapy resistance in various cancers. Multiple genes, such as p53, may be involved in the radioresistance and chemoresistance of cancer cells. For example, mutations in the p53 gene are found in more than 50% of malignant tumors (the most common genetic change in tumors), indicating that mutation in this gene is likely to be closely related to the generation of resistance.^272^ According to clinical data, a deletion mutation of p53 is associated with prolonged survival of esophageal cancer patients.^273^ The radioresistance and chemoresistance status of cancer is based on many complex factors and mechanisms. This review provides a reference for cancer therapy and the sensitivity of tumor cells to radiotherapy or chemotherapy.

**FIGURE 4:**
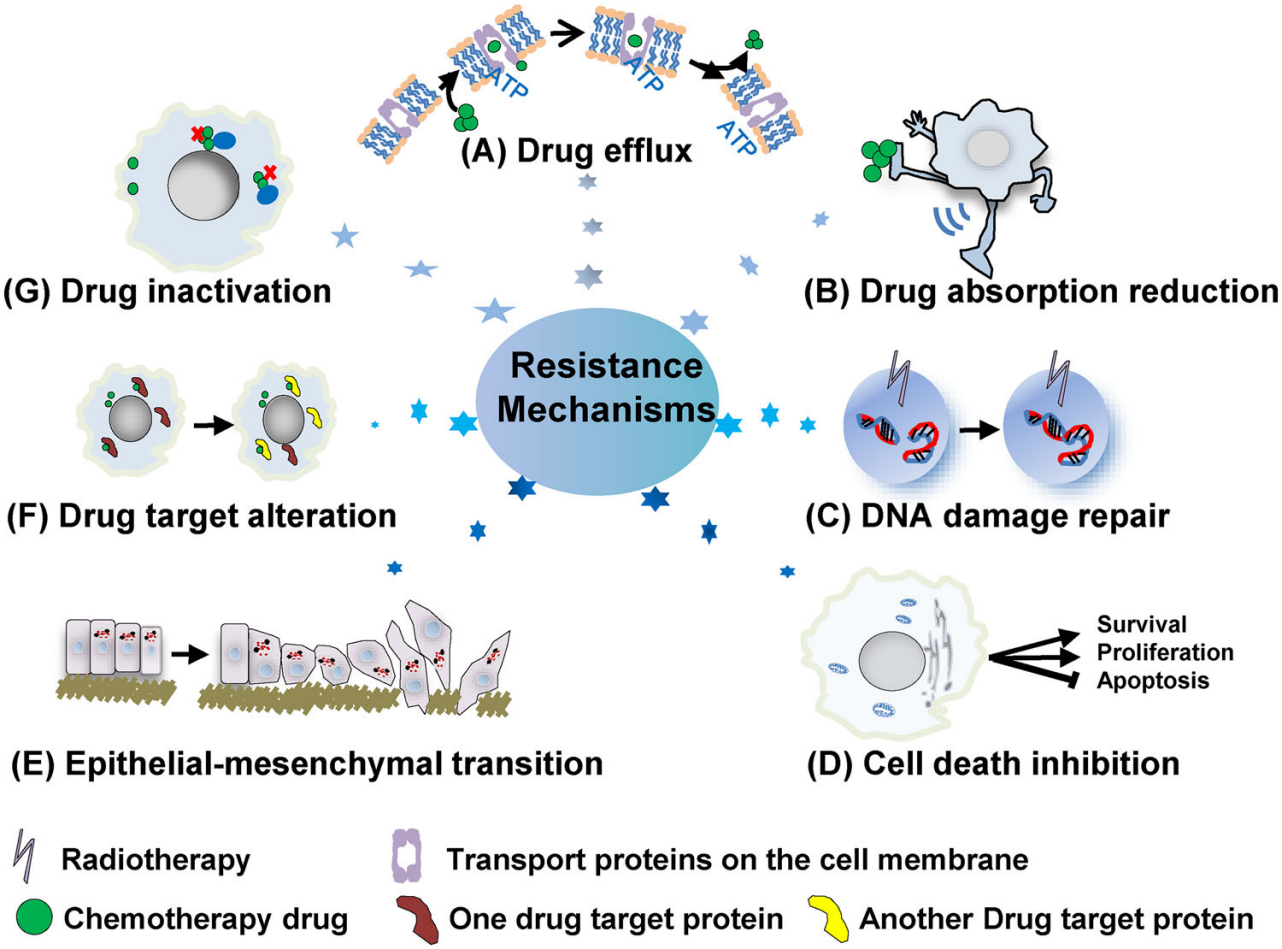
Molecular mechanisms of tumor chemoresistance and radioresistance. (A) Abnormal expression of ABC family contribute to drugs being pumped out of the cell, which causes intracellular drug concentrations too low to sensitive to drugs. (B) Changes in the expression of transport proteins in drug absorption lead to a decreased drug absorption rate and chemoresistance. (C) DNA damaged by chemotherapy and radiotherapy is repaired quickly, which is closely related to the acquisition of chemoresistance and radioresistance. (D) Cell death is inhibited, indicating that the balance between apoptosis and cell growth is disrupted, affected by the major gene families such as p53 and Bcl. (E) The production of cellular EMT properties causes resistance to chemotherapy and radiotherapy. (F) The molecular targets of drugs can be altered in tumor cells. (G) Drug inactivation, some detoxification‐related proteins deactivate drugs in cells, which is followed by chemoresistance acquisition

Taken together, therapy resistance in cancer is a complex process, and multiple mechanisms of chemoresistance and radioresistance were discovered in cancer cells (Figure 4), and it is necessary to conduct an in‐depth study on the genetic basis of chemotherapy or radiotherapy resistance in cancer to establish new treatment methods that may help resolve clinical treatment failure, which will reduce the recurrence and metastasis of the tumor.^2^, ^8^, ^274^ The results from the study of genes related to resistance to chemoradiotherapy may be useful for determining prognostic indicators and targets for the molecular treatment of cancer.

## AUTHOR'S CONTRIBUTIONS

Ya‐Ping Liu, Can‐Can Zheng, Yun‐Na Huang and Ming‐Liang He wrote the manuscript. Bin Li and Wen Wen Xu designed and revised the manuscript. All authors commented on the manuscript and approved the final manuscript.

## ETHICS STATEMENT

Not applicable.

## CONFLICT OF INTEREST

The authors declare no conflict of interest. Author Ming‐Liang He is the Editorial Board Member of MedComm. Author Ming‐Liang He was not involved in the journal's review of, or decisions related to, this manuscript.

## Data Availability

Not applicable.
